# The Effect of Iranian Customary Used Probiotic Yogurt on the Children’s Salivary Cariogenic Microflora

**Published:** 2015-06

**Authors:** Ali Nozari, Mohammad Motamedifar, Nasim Seifi, Zeynab Hatamizargaran, Mohammad Ali Ranjbar

**Affiliations:** 1 Dept. of Pediatric Dentistry, Shiraz University of Medical Sciences, Shiraz, Iran;; 2 Dept. of Bacteriology & Virology, Shiraz HIV/AIDS Research Center, School of Medincne, Shiraz University of Medical Sciences, Shiraz, Iran;; 3 Dept. of Pediatric Dentistry, School of Dentistry, Shiraz University of Medical Sciences, Shiraz, Iran;; 4 Dept. of Clinical Nutrition, School of Nutritional Sciences and Dietetics, Tehran University of Medical Sciences, Tehran, Iran;; 5 Dept. of Oral and Maxillofacial Pathology, Shiraz University of Medical Sciences, Shiraz, Iran;

**Keywords:** Probiotics, Tooth caries, Streptococcus mutans, Lactobacilli, Bifidobacterium lactis

## Abstract

**Statement of the Problem:**

Dental caries is the most common disease of childhood. Using probiotics has recently been introduced to reduce the incidence of dental caries.It consists of live microbial food supplements that beneficially affect the host, and hence are considered an alternative way to eradicate the infections.

**Purpose:**

The aim of this study is to evaluate the effect of consumption of probiotic yogurt on the children’s salivary cariogenic microflora.

**Materials and Method:**

A double-blind randomized study was performed recruiting 2 parallel groups; 24 healthy children in the case and 25 children in the control group. All healthy children were followed- up over 4 periods. Periods 1 and 3 were wash-out periods with duration of 1 and 2 weeks, respectively. During periods 2 and 4 (2weeks duration each), the case group consumed 200g yogurt containing *Bifidobacterium lactis* (1×10^6 ^per gram) once daily and the control group consumed normal yogurt. Salivary *Streptococci mutans* and *Lactobacilli* were enumerated before and after the yogurt consumption periods. Pre- and post-treatment values within and between regimens were compared using the t-test and paired samples.

**Results:**

There was a reduction in *Streptococcus mutans* and *Lactobacillus* counts in the control group, but for *Streptococcus mutans*, the count reduction between phases 1 and 4 was statistically significant (*p*= 0.009). In the case group, neither the *Streptococcus mutans *count nor the *Lactobacilli* count was significantly reduced.

**Conclusion:**

Based on the findings of this study, short-term daily consumption of probiotic yogurt containing *Bifidobacterium lactis* could not reduce the levels of salivary *Streptococcus mutans* and *Lactobacilli* in 6 to 12 year-old children, while normal yogurt could reduce the *Streptococcus mutans* counts significantly.

## Introduction

Dental caries is the most common chronic disease affecting the majority of adults and 60-90% of school children.[1] Caries is a multifactorial disease with bacterial origin that can demineralize tooth enamel.[2] In dental caries, there is an increase in acidogenic and acid-tolerating species such as *mutans Streptococci* and *Lactobacilli,* although other bacteria can be found. Changes in the homeostasis of the oral cavity would enhance proliferation of bacterial biofilm, notably mutans, from the streptococci group.[3]

*Streptococcus mutans* is believed to play an important role in the initiation of caries and *Lactobacillus *to have a part in the progression of tooth decay.[4] One of the methods that have recently been considered effective on the reduction of dental caries and decrement of cariogenic flora is the use of probiotic products.[5] Probiotics can promote oral health and also prevent oral diseases like caries, gingivitis, periodontitis and halitosis. The use of health promoting bacteria is one of the novel approaches in oral health.[5] The consumption of probiotics may have a favorable effect on the reduction of dental caries.[6-7]

Several studies have shown that using the probiotic products could replace and ex-change the cariogenic bacteria with non-cariogenic ones.[8] These studies have employed different approaches. One approach is making use of the ability of probiotic bacteria to colonize on teeth and influence the supragingival plaque.[8] Probiotics must be able to attach to the surface of teeth and integrate into the bacterial population, making the dental biofilm.[5]

The term probiotic, introduced by Gibson and Roberfroid, is a live non-digestible microbial food whose efficacy on human health has been established.[6] A number of probiotic products that can play a critical role in human health have been proposed to have the potential to improve intestinal balance and influence the immune system through molecular mechanisms.[9-11]

Other studies have investigated the application of probiotics in treating cardiovascular diseases, urogenital infections and cancer[12-15] but little attention is paid to its possible effect on oral health and risk factors of caries. The present study has focused on the effect of probiotics on oral microorganisms.

Nase *et al.* (2001) investigated the effect of probiotic *Lactobacillus* *rhamnosus *on caries risk and showed less caries and lower *Streptococcus mutans* levels in probiotic-milk consuming group.[16] Caglar *et al.* (2007) estimated the efficacy of Xylitol and probiotic chewing gums on the count of *Streptococcus mutans* and *Lactobacilli* and reported that probiotic bacteria could reduce the salivary level of *Streptococcus mutans* significantly.[17]

Steckson-Blicks *et al.* (2009) reported that daily use of probiotic milk and fluoride decreases the caries in pre-school children.[18] Moreover, Caglar *et al.* (2006) investigated the effect of the probiotic bacterium *Lactobacillus reateri ATCC 5573 *on salivary *mutans streptococci* and *Lactobacilli* and demonstrated that a short-term consumption of *Lactobacilli*-derived probiotics reduced the level of salivary *mutans streptococci* in young adults.[19] In another study, Singh *et al.* (2011) showed that probiotic ice-cream could decrease the count of *Streptococcus mutans*, but its effect on *Lactobacilli* count has not been significant.[20]

Regarding the findings of some studies that have demonstrated the probiotics to be useful in reducing the cariogenic flora of mouth and also the availability of probiotic yogurt, the present study is aimed to evaluate the effect of probiotic yogurt consumption on the cariogenic flora of children. We were also interested to explain whether the effect of probiotics would remain during the predefined four different periods. 

## Materials and Method

This randomized double-blind study recruited 49 healthy children with the age range of 6-12 years old (mean age=9.2±1.7) referred to the School of Dentistry, Shiraz University of Medical Sciences. Participants had not used any probiotic products. Those who were receiving Xylitol-containing products or fluoride treatments were excluded from the study. Using any fluoride products was forbidden.

Participants were instructed to brush their teeth twice a day and maintain their normal oral hygiene. Using chewing gums containing Xylitol as well as probiotic yogurt during the study was forbidden. Any changes in health and using any medicine were considered. An experienced dentist examined the oral health of the participants before the study.

This study had two parallel groups; 24 healthy children in the case group and 25 healthy children in the control group. Both groups were matched on the following attributes: age, gender and DMFT. All of the children in the study were followed up over four periods. Periods 1 and 3 were wash-out periods with duration of 1 and 2 weeks, respectively. During periods 2 and 4 (2 weeks each), the participants were asked to eat 200g yogurt, containing *Bifidobacterium lactis,* 10[6] colony forming units (CFU)/mL, once daily at lunch. Tooth brushing was forbidden for at least 1 hour after eating probiotic yogurt. Since the most frequently available probiotic bacteria are *Lactobacillus* and *Bifidobacterium*,[21] we used a probiotic yogurt containing *Bifidobacteriu* in this study.

Sampling of saliva was done after each phase and the participants were prevented from eating and drinking 3 hours before sampling. Then, 5mL non-stimulated saliva was collected in a sterile graded test tube (Falcon; China). The colonies of streptococci mutans were counted after culture and incubation period of 48 hours in blood agar (Merk; UK) plates at 37˚C. Meanwhile, lactobacilli were cultured in tomato juice agar (Himedia, India) anerobically and their colonies were counted with a stereomicroscope (Zeiss; German) after an incubation period of 48 hours at 37°C. Statistical analysis was carried out using SPSS Software, version 14.0. Data were analyzed using t-test and paired- samples method. Differences were considered significant at *p*< 0.05.

## Results

**Figure 1:**
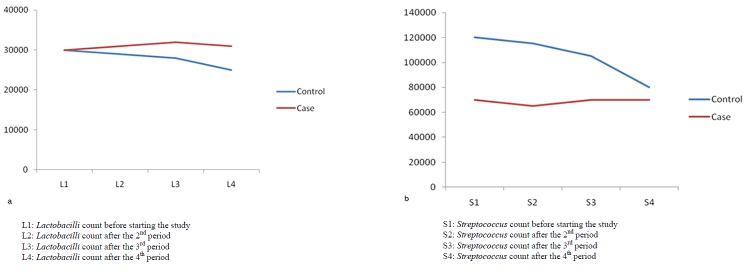
: Changes *in Lactobacilli* count

Figures 1 illustrate the levels of *Streptococcus mutans* and *Lactobacilli* both case and control groups in different phases. As shown in the Figures, from phase 1 to phase 4, there was no significant difference in *Lactobacilli *counts between case and control groups (*p*= 0.383); while the *Streptococcus mutans* counts exhibited a statistically significant difference between the two groups (*p*= 0.003). Although there was a reduction in *Streptococcus mutans* and *Lactobacillus* counts between phases 1 and 4 in control group, the count reduction was significant and prominent only for *Streptococcus mutans* (*p*= 0.009). In the case group, neither the *Streptococcus mutans* count nor the *Lactobacilli* count was significantly reduced after consumption of probiotic yogurt.

Normal yogurt decreased the *Streptococcus mutans* level significantly between period 1 and 2, also between period 3 and 4, but probiotic yogurt could not reduce the *Streptococcus mutans* count between these periods. Distributions of salivary mutans streptococci and *Lactobacilli* in the case and control groups are summarized in Table 1 and 2.

**Table 1:** Distribution of salivary mutans streptococci and lactobacilli counts at the end of 4 different periods in the case group

	**S1**	**S2**	**S3**	**S4**	**L1**	**L2**	**L3**	**L4**
Number of cases	24	24	24	24	24	24	24	24
Mean count of microorganisms	60083	49750	55416	50416	32416	33451	34666	32452
SD	40696	32647	35604	30684	16895	18996	20580	31112
Minimum count of microorganism	0	0	0	0	0	0	0	0
Maximum count of microorganism	200000	120000	140000	100000	60000	80000	100000	160000
S1: streptococcus count after the 1^st^ period S2: streptococcus count after the 2^nd^ period S3: streptococcus countafter the 3^rd^ period S4: streptococcus count after the 4^th^ period L1: Lactobacilli count after the 1^st^ period L2: Lactobacilli count after the 2^nd^ period L3: Lactobacilli count after the 3^rd^ period L4: Lactobacilli count after the 4^th^ period								

**Table 2:** Distribution of salivary mutans streptococci and lactobacilli counts at the end of 4 different periods in the control group

	**S1**	**S2**	**S3**	**S4**	**L1**	**L2**	**L3**	**L4**
Number of cases	25	25	25	25	25	25	25	25
Mean count l of microorganism	129200	120160	112080	64960	33120	30000	26080	23040
SD	97891.1	89489.1	84418.1	62198.9	18412.7	15874.5	15721.3	14249.2
Minimum l count of microorganism	0	0	0	0	0	0	0	0
Maximum count of microorganism	360000	340000	360000	240000	40000	70000	60000	80000
S1: streptococcus count after the 1^st^ period S2: streptococcus count after the 2^nd^ period S3: streptococcus count after the 3^rd^ period S4: streptococcus count after the 4^th^ period L1: Lactobacilli count after the 1^st^ period L2: Lactobacilli count after the 2^nd^ period L3: Lactobacilli count after the 3^rd^ period L4: Lactobacilli count after the 4^th^ period								

## Discussion

The findings of this study showed that short-term consumption of probiotic yogurt could decrease neither *Streptococcus mutans* nor *Lactobacillus* counts. Caglar *et al.* (2009) investigated the colonization of *Lactobacillus reuteri* *ATCC 55730* in the oral cavity after discontinuing the administration of probiotic product prepared with this bacterium.[22] The results showed that *Lactobacillus* reuteri had not been colonized permanently in the oral cavity after a period of 2 weeks consumption.

In accordance with most studies conducted before, this study showed no reduction of salivary *Lactobacilli *count after consumption of probiotic yogurt. There was even an increase in *Lactobacilli* growth in some cases. It can be concluded that the types of microorganisms in probiotic yogurt could not decrease and compete with the oral normal *Lactobacilli* flora.

Ahola *et al.* (2002) reported that all samples within the probiotic group revealed even increased *Lactobacilli *counts.[23] In the present study, after a short term consumption of probiotic yogurt, there was no statistically significant reduction in *Streptococcus mutans* and *Lactobacilli *counts. Ahola *et al.* used Edam cheese, containing *Lactobacillus rhamnosus* GG and *Lactobacillus rhamnosus LC 7.5* to 18-35 year-old participants and found that the *Streptococcus mutans* count decreased in 20% of participants.[23] The difference between our findings and that of Ahola *et al.* may be explained by the difference of age range and the type of the probiotic bacteria.

In the control group, consumption of yogurt reduced the *Streptococcus mutans* and *Lactobacilli *count, but for *Streptococcus mutans*, the reduction was significant after period 4. The type of *Lactobacillus *present in the control group yogurts may be similar or almost similar to the oral *Lactobacilli* which can compete with them and hence reduce the member of oral normal flora of *Lactobacilli* to some degrees. Furthermore, the presence of some microbial products or other formulas found in control group yogurt, but not in the probiotic samples, might be responsible for the inhibition of the *Streptococcus mutans *proliferation. The type of probiotic microorganism used in the yogurt (*Bifidobacterium lactis*) did not have any inhibitory effect on *Streptococcus mutans* proliferation. Caglar *et al.* (2005) and Cildir *et al.* (2009) reported reduction of the salivary level of *Streptococcus mutans* after a short-term consumption of probiotic yogurt.[24-25] However, their studies differed from the present study, both in terms of the age range of participants and the type of probiotic bacteria used.

Our data revealed that usage of common yogurt could decrease bacterial level more than probiotic yogurt in case group. This finding is comparable with that of Petti *et al.* (2001) that demonstrated the effect of yogurt containing *L.bulgaricus* on the salivary microflora.[26] It suggests that normal yogurt can decrease the count of *mutans streptococci* and l*actobacilli* and can be used as a caries-preventing food.

In a systematic review, Cagetti *et al.* evaluated several papers about the use of probiotic strains in caries prevention.[27] This assessment revealed that all studies were generally small or medium in sample size and majority of them (80%) were short-term interventions. The quality of published papers recorded using the Consort score was 4 excellent, 9 good and 10 poor. These evidences have limited the results obtained about the efficacy of probiotics usage in caries prevention. Although administration of probiotics for prevention of caries has ascertained promising results, only few studies have shown clear clinical outcome; hence, the scientific data is still poor.

Owing to brevity of consumption period of probiotic product in this study and the limitation of probiotic microorganism type, more investigations are required to show the effect of longer consumption period of probiotic products on the salivary level of *streptococcus mutans*. Until the time of this study, none of the studies, conducted on the role of probiotic in caries prevention, have evaluated the effect of variable pH of probiotic yogurts on salivary cariogenic microflora, although changes of yogurt pH within the consumption period should be considered. Therefore, assessing the efficacy of different probiotic yogurt with variable pH on the risk factors of caries is recommended.

## Conclusion

Based on the findings of this study, short-term daily consumption of probiotic yogurt containing *Bifidobacterium lactis* could not reduce the levels of *salivary streptococci* mutans and *lactobacilli* in 6 to 12 year-old children while the normal yogurt could significantly reduce the number of *streptococcus mutans.*
